# An Antisense Long Non-Coding RNA, LncRsn, Is Involved in Sexual Reproduction and Full Virulence in *Fusarium graminearum*

**DOI:** 10.3390/jof10100692

**Published:** 2024-10-03

**Authors:** Zhizhen Fu, Yanjie Chen, Gaolei Cai, Huijuan Peng, Xiaoyu Wang, Ping Li, Aiguo Gu, Yanli Li, Dongfang Ma

**Affiliations:** 1 Key Laboratory of Sustainable Crop Production in the Middle Reaches of the Yangtze River, College of Agriculture, Yangtze University, Jingzhou 434025, China; 2022720883@yangtzeu.edu.cn (Z.F.); 2022710812@yangtzeu.edu.cn (Y.C.); 2024720966@yangtzeu.edu.cn (H.P.); 2023710865@yangtzeu.edu.cn (X.W.); 2022710831@yangtzeu.edu.cn (P.L.); 2 Shiyan Academy of Agricultural Sciences, Shiyan 442000, China; caigaolei@163.com; 3 Jiangsu Product Quality Testing & Inspection Institute, 5 Guanghua Street, Nanjing 210007, China; nj180618@163.com

**Keywords:** *Fusarium graminearum*, long non-coding RNA, sexual reproduction, full virulence, *FgSna*

## Abstract

Fusarium head blight (FHB), primarily caused by *Fusarium graminearum*, is a devastating crop disease that leads to significant declines in wheat yield and quality worldwide. Long non-coding RNAs (lncRNAs) are found to play significant functions in various biological processes, but their regulatory functions in the sexual reproduction and pathogenicity of *F. graminearum* have not been studied extensively. This study identified an antisense lncRNA, named lncRsn, located in the transcription initiation site region between the 5′-flanking gene *FgSna* and the 3′-flanking gene *FgPta*. A deletion mutant of lncRsn (ΔlncRsn) was constructed through homologous recombination. ΔlncRsn exhibited huge reductions in pathogen and sexual reproduction. Additionally, the deletion of lncRsn disrupted the biosynthesis of deoxynivalenol (DON) and impaired the formation of infection structures. RT-qPCR analysis reveals that lncRsn may negatively regulate the transcription of the target gene FgSna. This study found that lncRsn plays an important role in sexual and asexual reproduction, pathogenicity, virulence, osmotic stress, and cell wall integrity (CWI) in *F. graminearum*. Further characterization of pathogenesis-related genes and the reaction between lncRsn and protein-coding genes will aid in developing novel approaches for controlling *F. graminearum* diseases.

## 1. Introduction

Long non-coding RNAs (lncRNAs) are transcripts longer than 200 bp that have no protein-coding function [1]. These molecules have been shown to play significant roles in diverse biological processes [2,3]. In the genomes of higher organisms, the majority of lncRNAs are primarily transcribed by RNA polymerase II. Under some specific conditions, small amounts of lncRNAs can encode functional oligopeptides but with very low coding capacity [4]. Compared with protein-coding genes, lncRNAs are less evolutionarily conserved and are not highly expressed. However, they exhibit spatial and temporal specificity in different tissues and are important components of the gene regulatory network. LncRNAs are transcribed from the antisense or sense strands of coding genes. Based on their genomic location, lncRNAs are typically transcribed from intronic, intergenic, antisense, or sense regions of coding genes. Based on their genomic location, lncRNAs are classified into three groups: incRNAs, NATs, and lincRNAs [5,6,7]. These lncRNAs play important functions in various physiological and pathological processes and have recently emerged as major regulators of the transcriptional process.

LncRNAs were initially discovered in humans but have since been found to play important functions in various cellular processes across many eukaryotic organisms. While numerous lncRNAs have been well-characterized in mammals, cancer cells, and plants [8,9,10], our understanding of lncRNAs in filamentous fungi remains quite limited, particularly in the case of the plant pathogen *Fusarium graminearum*. To date, the presence of lncRNAs has only been predicted in a handful of fungal species, including the industrially relevant *Trichoderma reesei*, model organism *Neurospora crassa*, fission *Schizosaccharomyces pombe*, the green alga *Chlamydomonas reinhardtii*, smut fungus *Ustilago maydis*, and several Aspergillus and Colletotrichum (anthracnose) species, in addition to *F. graminearum*. In *T. reesei*, the lncRNA HAX1 shows a positive influence on the expression of cellulase-encoding genes by interfering with the negative feedback regulation of the transcription factor Xyr1 through a complex mechanism [11]. Meanwhile, in *N. crassa*, while lncRNAs and natural antisense transcripts (NATs) have been annotated and characterized through RNA sequencing, their specific regulatory functions remain unclear [12,13]. The role of lncRNAs is better understood in the fission yeast *S. pombe*. This study found that lncRNA nam1 is targeted by the RNA-binding protein Mmi1, which triggers its degradation by nuclear exosomes. This process also mediates the transcriptional termination of nam1, preventing the downstream genes from functioning. Mmi1-dependent termination of lncRNAs also occurs in the peripheral centromeric regions, contributing to heterochromatin formation and gene silencing, together with RNA interference mechanisms [14]. In *S. pombe*, recessive intron-containing lncRNAs are also associated with splicing factors through the conserved Pir2ARS2 protein, which recruits RNA processing and chromatin modification activities to mediate gene repression [15]. The RNA interference (RNAi) pathway in *F. graminearum* enables the natural antisense non-coding RNA known as *GzmetE-AS* to suppress the expression of the *GzmetE* gene. This suppression plays a significant function in regulating both asexual and sexual reproduction in *F. graminearum* [16]. Additionally, when the full-length antisense transcript RNA5P is overexpressed in its natural environment, it inhibits the expression of the key toxigenic gene TRI5, leading to reduced production of the mycotoxin DON [17]. These findings indicate that lncRNAs are involved in various biological processes within filamentous fungal cells, particularly in their pathogenic mechanisms. However, our current understanding of lncRNA function and regulatory mechanism in these organisms remains very limited. Further research is needed to investigate the mechanisms by which lncRNAs contribute to fungal growth, development, and pathogenicity.

Fusarium head blight (FHB) is globally widespread and spreads rapidly in hot and humid climates, leading to significant reductions in wheat yield and quality [16,17,18,19]. During the sexual reproduction stage of its life cycle, *F. graminearum* produces ascospores, which aid in fungus survival through winter and allow it to parasitize dead grass plants [19]. In the following spring, the fungus develops mycelium that spreads via conidia to infect healthy wheat plants. Once a wheat plant is infected, *F. graminearum* causes the breakdown of starchy materials and proteins within the grain, leading to the synthesis of the dangerous DON toxin. This results in the development of brown lesions on the infected wheat within a few weeks [20]. The DON toxin accumulates in the wheat kernels and causes a serious threat to food quality and safety for both humans and animals [21,22,23]. The production of DON in *F. graminearum* is linked to the *T* gene cluster [24]. While some microorganisms that can degrade DON have been discovered, their practical application in agriculture remains limited. The current management of FHB primarily relies on the use of certain fungicides and pesticides [19]. Given this scenario, there is an urgent need for the development of new, less toxic, and effective fungicides to combat this devastating fungal disease.

In the genome of *F. graminearum*, there is an antisense lncRNA located between two protein-coding genes, *FGSG_09021* and *FGSG_09020*, in their transcription termination regions. The expression levels of *FgSna* (*FGSG_09021*) and *FgPta* (*FGSG_09020*) were observed to be low during the infestation period compared with the nutrient mycelium period. In contrast, the expression level of the antisense lncRNA (lncRsn) was found to be high during the infestation period compared with the nutrient mycelium stage. This pattern of differential expression caught our attention and prompted further investigation into the functional roles of this lncRNA. To study the functions of lncRsn, we obtained lncRsn deletion mutants through the process of homologous recombination. This was facilitated by the use of polyethylene glycol (PEG)-mediated knockout of the gene sequence where the lncRsn is located. This study aims to examine the functional roles of lncRsn in various aspects of the fungus’s biology, including nutrition for growth, asexual and sexual development, phytopathogenic infection, and other relevant traits.

## 2. Materials and Methods

### 2.1. Fungal Strains and Culture Conditions

The *F. graminearum* wild-type (WT) strain PH-1 used in this study was kindly provided by Dr. Huaigu Chen from the Jiangsu Academy of Agricultural Sciences. All mutant strains were generated using the split-marker approach as previously described [25], with primers 1F, 2R, 3F, and 4R (Table S1). PH-1 and all mutant strains were cultured on potato sucrose agar (PSA) plates for 3–4 days at 25 °C. The colony morphology, growth rate, and conidial germination were measured on PSA and complete agar medium (CM) plates incubated at 25 °C.

In this study, three mycelial plugs of each strain were placed in a 50 mL carboxymethyl cellulose CMC liquid medium and incubated at 25 °C in a shaker (150 rpm) for 5 days. The number of conidia produced by each strain was quantified using a hemocytometer. The harvested conidia were then inoculated into yeast extract peptone dextrose (YEPD) liquid medium and incubated at 25 °C in a shaker (200 rpm) for 0, 6, and 12 h. Conidial germination was examined using differential interference contrast (DIC) microscopy or calcofluor white (CFW) staining.

### 2.2. Generation and Detection of Mutant Strains 

Mycelium was collected from overnight cultures of YEPD liquid medium (incubated for 12–16 h) using sterile filter paper. The mycelium was then washed with sterile water, and the osmotic pressure of the mycelium and protoplasts was maintained using 1.2 M KCl. The mycelium was enzymatically digested for 2.5 h using a ready-made enzyme solution mixture buffer (containing 2% Lysozyme, 3% Snailase, 2% Cellulase, and 0.5% Lysing Enzymes in 10 mL KCl). Filtration and centrifugation through the filter paper resulted in the isolation of protoplasts.

The protoplasts were washed once with STC buffer, centrifuged, and then resuspended in STC at a concentration of 1 × 10^8^ protoplasts/mL. A total of 30 to 50 μg of DNA was used to transform the wild-type *F. graminearum* strain PH-1 protoplasts. The regenerating protoplasts were plated on a two-layer TB3 agar medium, with the bottom layer containing 200 μg/mL of hygromycin B and 50 μg/mL of ampicillin and the top layer containing 250 μg/mL of hygromycin B. The transformants were then screened by PCR using the primer pairs 5F+6R, H850+H852, 7F+H856R, and H855F+8R to grossly confirm the deletion of lncRsn.

### 2.3. RT-qPCR Detection of lncRsn and Its Neighboring Gene

Fluorescence quantitative RT-qPCR was used to detect the expression level of lncRsn in the mutant. Samples were cultured in a YEPD liquid medium for 24 h in the dark, and the resulting cDNA was used as a template for the RT-qPCR analysis. The primer pairs qlncRsn-F and qlncRsn-R (Table S1) were used to amplify lncRsn. For the pathogenicity stage, total RNA samples were extracted from wild-type *F. graminearum*-infected wheat heads (cultivar Yangmai 20) at 4 and 8 dpi (days post-infection). Total RNA was extracted using a TRIzol reagent. First-strand cDNA was obtained using the HiScript IV RT SuperMix for qPCR (+gDNA wiper) and oligo(dT) primers. *FGSG_09530* was used as an internal control with the primers qTublin-F + qTublin-R (Table S1). The expression of the *FgSna* and *FgPta* was analyzed using the primer pairs *qFgSna-F* and *qFgSna-R* and *qFgPta-F* and *qFgPta-R*, respectively (Table S1). Then, the RT-PCR analysis was performed with the CFX 96 Real-Time PCR System (Bio-Rad, Hercules, CA, USA). The RT-qPCR analysis was performed in triplicate, and the fold changes during the nutrient period and the pathogenic period were calculated using the 2^−ΔΔCt^ method.

### 2.4. Plant Infection and DON Production Assays

Pathogenicity tests were carried out on wheat coleoptiles, wheat spikelets, and corn silks, following established protocols [26,27,28,29]. Samples from 5-day-old cultures grown on carboxymethyl cellulose (CMC) medium were used for the wheat coleoptiles and spikelets. Each strain was replicated 20–30 times using 3-day-old wheat seedlings in a growth chamber. For the wheat spike assay, a 10 μL suspension containing 1 × 10^6^ conidia/mL was applied to the flowering wheat spikes, specifically targeting the fifth kernel from the bottom [30]. Observations of pathogenicity were made 14 days post-inoculation, and the disease index for each strain was calculated according to standardized methods [31]. The virulence of *F. graminearum* on corn silks was assessed at 5 days post-inoculation (dpi). To stimulate toxin production in vitro, each strain was grown in a liquid medium designed for trichothecene biosynthesis induction (TBI) at 28 °C [32]. A deoxynivalenol (DON) production assay was performed in TBI cultures using a competitive ELISA-based DON plate kit (Beacon Analytical Systems, Saco, ME, USA) as described [33]. Additionally, the liquid cultures were analyzed via high-performance liquid chromatography (HPLC) to measure the concentrations of DON and ergosterol.

### 2.5. Microscopic Examination

The strains were cultured on the wheat leaves for 24–48 h after inoculation. To observe the infection structure, infected leaves were observed with a scanning electron microscope (SEM) [34,35]. To determine the germination rate of conidia, spore suspensions cultured for 5 days in CMC were examined under a fluorescence microscope (Eclipse 90i, Nikon, Tokyo, Japan). Perithecium formation and ascospore discharge were examined using a microscope (Axio Zoom.V16, Carl Zeiss Microscopy GmbH, Jena, Germany). All colony growth morphologies grown on plates were captured with the aid of a Canon EOS 80D camera.

### 2.6. Sexual Reproduction Assays

The sexual development stages, including perithecium formation and ascospore discharge, were examined as previously described [16,36]. To induce perithecium formation and the ascospore discharge assay, each strain was inoculated onto a carrot medium and incubated for 7 days [37]. The perithecium-bearing surface was oriented perpendicular to the slide surface, and the assemblies were incubated in a humidity chamber for 18–24 h to allow for ascospore release. To quantify the discharged ascospores, the carrot agar blocks were placed upside down for 24 h, allowing the mature perithecia to release ascospores onto the lids of petri dishes. The ascospores were harvested, and the number of ascospores was counted using a microscope and a hemocytometer. Each experiment was performed using three biological replicates.

### 2.7. Assays for Defects in Responses to Different Stresses and Cell Wall Integrity Analysis

A hyphal plug was taken from the edge of a colony that had grown on PSA for three days and inoculated on a plate of PSA medium containing 0.5 M CaCl_2_, 0.2 M MgCl_2_, 1.2 M NaCl, 1 M KCl, and 15 mM H_2_O_2_. This experiment was performed using three biological replicates. To explore whether the lncRsn plays a function in maintaining cell wall integrity or in osmotic regulation in *F. graminearum*, the gene deletion mutant strains and the wild-type strain were grown on complete medium CM plates with 0.05% SDS (*w*/*v*) and Congo Red (CR) (*w*/*v*). Additionally, their sensitivities to 0.05% H_2_O_2_ (oxidative stress) (*w*/*v*) were tested [38].

## 3. Results

### 3.1. Analysis of lncRsn, FgSna and FgPta

**Figure 1:**
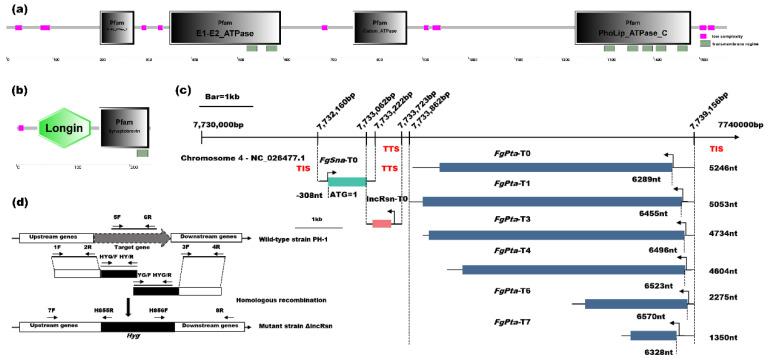
Identification and analysis of lncRsn, and its flanking genes *FgSna*, and *FgPta*. (**a**) Analysis of the FgPta protein sequence (1559 aa) reveals the presence of a PhoLip_ATPase_N domain (202–276 aa, grey), an E1-E2_ATPase domain (353–593 aa, grey), a Cation_ATPase domain (750–867 aa, grey), a PhoLip_ATPase_N domain, seven transmembrane helices (green); (**b**) Analysis of the *FgSna* protein sequence (236 aa) shows the presence of a Longin domain and has one transmembrane helix (green); (**c**) Strategy and identification of replacement of lncRsn with hygromycin gene (*Hygr* gene) in the wild-type strain PH-1 of *F. graminearum*. (**d**) The genomic location of lncRsn and the transcript isoforms of *FgSna* and *FgPta*. There are six transcript isoforms of *FgPta* based on their transcript lengths. *FgSna* has one transcript isoform. TIS: translation initiation site; TTS: transcription termination site.

Based on the previous study [39], an antisense lncRNA was detected between *FGSG_09021* and *FGSG_09020* in the RNA sequencing. NCBI domain analysis indicated that *FGSG_09021* encodes an uncharacterized protein belonging to the synaptobrevin family. The *FGSG_09021* gene was found to encode a protein consisting of 236 amino acids (http://fungi.ensembl.org/Fusarium_graminearum/Info/Index) (accessd on 10 October 2023), with one transmembrane helix region (213–232 aa) according to the TMHMM v2.0 program and a homolog of the synaptobrevin pfam domain (146–234 aa) as per the SMART protein database (http://smart.embl-heidelberg.de) (accessd on 10 October 2023) (Figure 1b). Therefore, *FGSG_09021* was designated as *FgSna*, and the antisense lncRNA was named lncRsn. *FGSG_09020* is located 138 bp downstream of lncRsn in the same direction. Based on the TMHMM v2.0 (http://www.cbs.dtu.dk/services/TMHMM/) (accessd on 10 October 2023) transmembrane domain prediction, it possesses seven transmembrane helices (Figure 1a). *FGSG_09020* (1559 aa) possesses seven transmembrane helices (Figure 1a). *FGSG_09020* is a phospholipid-transporting ATPase at the N and C termini, belonging to the cation transport ATPase (P-type) family, and was named *FgPta* for convenience. *FgSna* and lncRsn both have one transcript, named *FgSna*-T0 and lncRsn-T0. *FgSna*-T0 is 1063 nt and overlaps with *lncRsn*-T0 by 160 bases. There are six transcript isoforms of *FgPta*, named *FgPta*-T0 (5246 nt), *FgPta*-T1 (5053 nt), *FgPta*-T3 (4734 nt), *FgPta*-T4 (4604 nt), *FgPta*-T6 (2275 nt), and *FgPta*-T7 (1350 nt), based on the length of the transcript (Figure 1c). Relative to the start codon of *FgSna*, which is designated as position 1, the transcription start site of the target gene *FgPta* is situated at 108 nt. All transcripts of *FgPta* are in the opposite direction to that of *FgSna*-T0 and are located at 5246 nt, 5053 nt, 4734 nt, 4604 nt, 2275 nt, and 1350 nt, respectively. *FgPta*-T1, *FgPta*-T3, *FgPta*-T4, *FgPta*-T6, and *FgPta*-T7 have been expressed at extremely low levels in various periods, suggesting that *FgPta* is mainly regulated by *FgPta*-T0, so we do not consider the effect of the other five transcripts on *FgPta*.

Transcriptome data of *F. graminearum* showed that lncRsn was up-regulated during the fungus’s infestation of wheat and sexual reproduction compared with the vegetative hyphae growth period (Table S2). This suggests that lncRsn may play a role in regulating *F. graminearum* pathogenicity and sexual reproduction. Further analysis using the Coding-Non-Coding Index (CNCI), Coding Potential Calculator (CPC), and PLEK (Predictor of Long non-coding RNAs) [40,41,42] revealed that lncRsn sequences do not have the ability to encode proteins (Table S3). To investigate the impact of lncRsn on the pathogenicity and sexual reproduction of *F. graminearum*, the lncRsn gene was knocked out using homologous recombination. The entire lncRsn-T0 transcriptional region (7733062–7733723, 662nt of Chromosome 4) was deleted from PH-1 and replaced by the hygromycin gene (*Hygr* gene) (Figure 1d).

### 3.2. lncRsn Is Essential for Sexual Reproduction in F. graminearum

**Figure 2:**
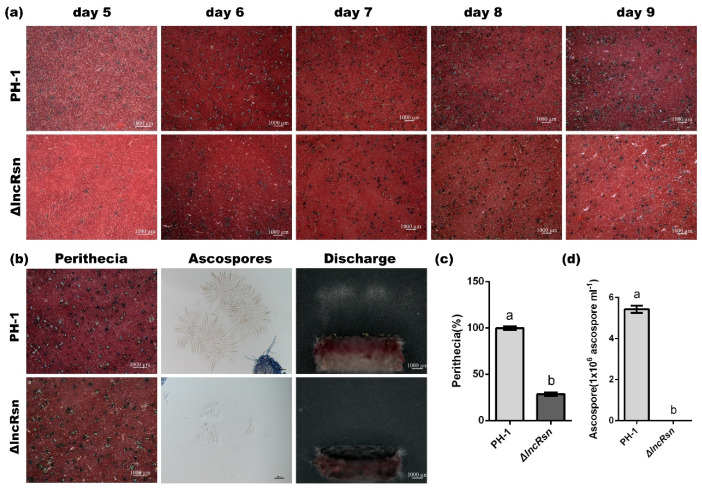
LncRsn is essential for asexual reproduction in *F. graminearum*. (**a**) Morphology of the sexual fruiting bodies (perithecia) produced by the wild-type PH-1 strain and the lncRsn deletion mutant (ΔlncRsn) strain. Photographs were taken using a 1000 μm scale bar; (**b**) Perithecia, ascus formation, and ascospore discharge photographed at 14 dpi; (**c**) Quantification of the number of perithecia and the ratio of perithecia with appendages (whips) in the wild-type and mutant strains, (*p* < 0.01); (**d**) Ascospores from the different strains, significant differences between the replicates are represented by letter a and b.

The expression of lncRsn and *FgSna* was significantly up-regulated during the sexual reproduction period (Figure S1). To investigate the role of lncRsn in the sexual reproduction process of *F. graminearum*, we inoculated the wild-type strain PH-1 and the lncRsn knockout strain ΔlncRsn on carrot agar medium to induce sexual reproduction. Compared with the PH-1 strain, the number of perithecia produced by the mutant ΔlncRsn strain was reduced by 71.5% (28.5 ± 3.29% of the wild-type) (Figure 2c). This indicates a significant decrease in perithecial formation by the lncRsn knockout strain 14 days post-fertilization (dpf). As a consequence, the knockout of lncRsn also reduced the production of ascospores (sexual spores) (Figure 2b). Furthermore, the discharge of ascospores from the ΔlncRsn strain at 12–24 h was significantly lower, with almost no ascospores being discharged. When collected and sprayed with the 12–18 h accumulated ascospores, the concentration from the PH-1 strain was 5.43 ± 0.97 × 10^6^ spores/mL, consistent with previous studies, but the mutant ΔlncRsn strain had zero ascospore concentration (Figure 2b,d). Importantly, there was no significant difference between the ΔlncRsn strain and the PH-1 strain in the morphology of the ascospores (Figure 2b). These results suggest that the lncRsn plays a critical role in regulating the production and release of ascospores, the sexual spores crucial for the dissemination of *F. graminearum*. The knockout of lncRsn substantially impaired the sexual reproduction process of *F. graminearum*.

In order to clarify the role of lncRsn in the development of perithecia in *F. graminearum*, we observed the number of perithecia in different strains at 5, 6, 7, 8, and 9 dpf. This allowed for the determination of the time point at which perithecia were produced by the lncRsn knockout mutant strain ΔlncRsn. The results showed that the ΔlncRsn mutant strain began producing perithecia after 5 dpf (Figure 2a), indicating that lncRsn affects the development of perithecia in *F. graminearum* and, consequently, influences the sexual reproduction process of the fungus. The genes *MAT1-1-1*, *MAT1-1-2*, *MAT1-1-3*, and *MAT1-2-1* play important roles in the formation of ascospores and cystospores in *F*. *graminearum* [43]. To investigate their expression, we measured the levels of these genes in ascospores after 5 days of exposure to black light irradiation, using samples without irradiation as a control. The findings revealed significant differences in the expression of these four genes in the mutant strains ΔlncRsn compared with the wild-type strain PH-1 after irradiation. This suggests that the lncRNA lncRsn may regulate the expression of *MAT1-1-1*, *MAT1-1-2*, *MAT1-1-3*, and *MAT1-2-1* during sexual reproduction in *F. graminearum*, thereby influencing the formation of ascospore shells and the production of ascospores. The findings suggest that lncRsn plays a significant role in regulating sexual reproduction by impacting the development of perithecia in *F. graminearum*. Since perithecia are essential for the production and discharge of ascospores, the sexual spores are crucial for the disease cycle. These results demonstrate that lncRsn has a significant effect on the overall sexual reproduction and dissemination of *F. graminearum*.

### 3.3. lncRsn Is Essential for Pathogenicity in F. graminearum

**Figure 3:**
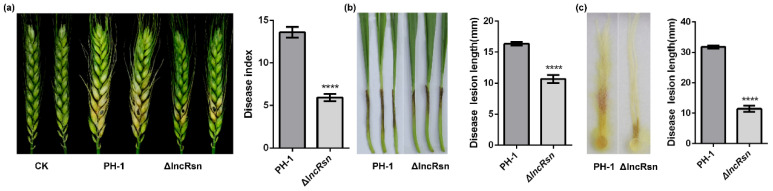
Roles of the lncRsn in fungal pathogenicity. (**a**) Wheat spikelets inoculated with each strain and observed at 14 days after inoculation; (**b**) Wheat coleoptiles inoculated with each strain and observed at 7 days after inoculation; (**c**) Corn silks were inoculated with each strain and observed at 5 days after inoculation, *p* < 0.01, significant differences between the replicates are represented by ****.

**Figure 4:**
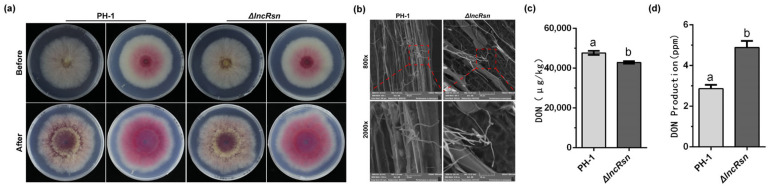
Roles of the lncRsn in mycelium penetration, infection structure, and DON production. (**a**) Mycelial penetration of wild-type PH-1 and ΔlncRsn strains on PSA medium after three days, with removal of aerial mycelium in cellophane after six days; (**b**) Infection structure formation of wild-type PH-1 and ΔlncRNA strains on wheat leaves after 24-48 h; (**c**) Analysis of DON toxin content in wheat kernels infected by different strains. (**d**) Analysis of DON toxin content of the strains in the culture supernatant incubated after seven days of TBI, *p* < 0.05, significant differences between the replicates are represented by letter a and b.

Pathogenicity assays were conducted on various wheat tissues, including flowering wheat heads, coleoptiles, and corn silks. On flowering wheat heads, the disease severity caused by the lncRsn deletion mutant strain was significantly lower compared with the wild-type strain. The disease index score for the mutant was approximately 5.9, indicating a substantial reduction in pathogenicity (Figure 3a). Similar results were observed on wheat coleoptiles. The wild-type strain PH-1 caused disease lesions that were 16.36 ± 0.31 mm in length, whereas the lncRsn mutant strain only caused 10.68 ± 0.64 mm of lesions. This represents a 34% decrease in virulence in wheat coleoptiles (Figure 3b). The trend continued on corn silks, where the wild-type PH-1 strain induced lesions of 31.78 ± 0.56 mm in length at 5 dpi. In contrast, the lncRsn mutant strain only caused 11.45 ± 0.99 mm lesions, a 63% reduction in virulence (Figure 3c). These results demonstrate that lncRsn plays a significant role in the pathogenicity of *F. graminearum* on various host tissues, including wheat and corn. Interestingly, the deletion of lncRsn did not significantly affect the ability of the fungus to penetrate plant tissues or its saprophytic growth on agar media (Figure 4a). Further microscopic analysis revealed that the lncRsn mutant strain formed smaller and less organized infection structures, such as cushions and invasive hyphae, on wheat leaves compared with the wild-type strain (Figure 4b). This indicates that lncRsn is important for the proper development of infection-related structures in *F. graminearum*. The findings suggest that the lncRsn gene is important in the pathogenicity of *F. graminearum*, likely by influencing the formation and function of infection structures without significantly affecting the fungus’s basic growth and penetration abilities.

### 3.4. Deletion of lncRsn Interferes with the Synthesis of DON

When *F. graminearum* infects wheat, it is capable of producing mycotoxins such as deoxynivalenol (DON) toxins and zearalenone (ZEA) toxins under suitable conditions and accumulating in wheat over a long period of time, which poses a serious threat to the health and safety of animals including human beings, as well as food safety [44]. Mycotoxins can significantly impact human and animal health, as well as food safety. Among these, DON is considered the most detrimental. To further investigate the role of lncRsn in regulating DON biosynthesis in *F. graminearum*, we analyzed DON levels in wheat kernels. We compared DON content after 7 days of in vitro induction and 14 days of infection with the wild-type strain versus a mutant strain lacking lncRsn (ΔlncRsn). The results showed that under in vitro induction, the wild-type strain produced 2.86 ± 0.19 ppb of DON, while the ΔlncRsn mutant had a significantly higher DON content of 4.88 ± 0.32 ppb (Figure 4d). However, in wheat kernels infected with the wild-type strain, DON levels reached 47,692.86 ± 1376.99 ppb, whereas in wheat infected with the ΔlncRsn mutant, DON was lower at 42,876.91 ± 818.18 ppb (Figure 4c). The expression levels of seven TIR gene clusters in toxin-producing corpuscles after 7 days of TBI culture were determined, and the results were consistent with the toxin detection results, indicating that lncRsn plays an important role in the regulation of TRI gene clusters (Figure S2). These results reveal that deleting the lncRsn gene disrupts the regulation of DON biosynthesis in *F. graminearum*, leading to a disturbance in the toxin synthesis process, suggesting that lncRsn plays a function in modulating DON production by this important plant pathogenic fungus.

### 3.5. Deletion of lncRsn Is Not Necessary for Asexual Reproduction but Display Reduced Sensitivity under Stress Conditions

**Figure 5:**
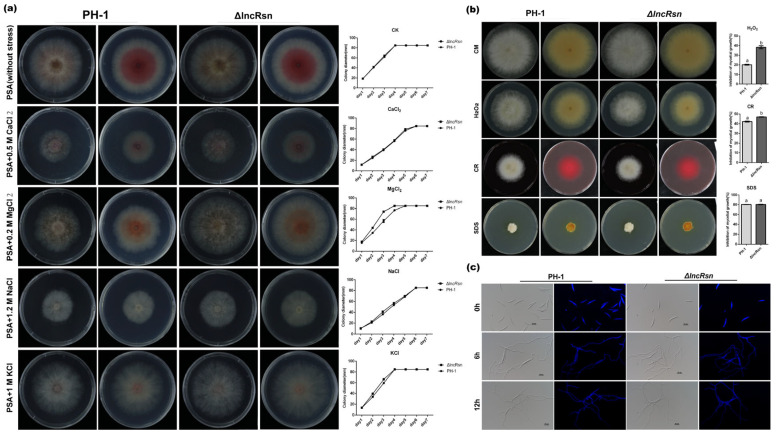
lncRsn is not necessary for asexual reproduction but displays sensitivity under stress conditions. (**a**) Colony morphology and diameter of PH-1 and ΔlncRsn on PSA medium; (**b**) Fungal growth assessment on CM plates with 0.05% (*w*/*v*) H_2_O_2_, Congo Red, and SDS. Stress growth inhibition rate analysis;(**c**) Conidial germination of lncRsn at a 25 µm scale bar, significant differences between the replicates are represented by letter a and b.

To investigate the role of lncRsn in response to *F. graminearumi* stress, we assessed mycelial growth in the presence of various osmotic agents. When grown on PSA and complete CM agar plates, the wild-type PH-1 and ΔlncRsn mutant strains displayed similar growth. However, on agar plates supplemented with osmotic agents (0.5 M CaCl_2_, 0.2 M MgCl_2_, 1.2 M NaCl, and 1 M KCl), the growth of both strains was significantly enhanced (Figure 5a). Compared with the wild-type PH-1 strain, the ΔlncRsn mutant exhibited significant resistance to MgCl_2_ and slight resistance to NaCl and KCl, though no other obvious defects were detected in the CaCl_2_ stress treatment assays.

The lack of chitin synthase or the cell wall integrity (CWI) pathway MAPK dramatically reduces the virulence of *F. graminearum* in host plants [44,45,46]. Therefore, we investigated how the gene deletion mutant strains respond to cell wall-damaging agents such as Congo Red (CR) and sodium dodecyl sulfate (SDS), as well as their sensitivity to oxidative stress induced by H_2_O_2_. As shown in Figure 5b, the ΔlncRsn strain displayed increased sensitivity to H_2_O_2_, CR, and SDS, which are common cell wall-damaging agents [46,47,48,49,50]. 

For conidial germination of the ΔlncRsn mutants in carboxymethyl cellulose (CMC) and yeast extract peptone dextrose (YEPD) media at 0 h, 6 h, and 12 h were examined (Figure 5c). No significant differences were observed in conidium production or conidial germination morphology compared with PH-1. These results indicate that lncRsn is not essential for asexual reproduction in *F. graminearum* but plays a role in maintaining cell wall integrity and osmotic regulation in this important plant, *F. graminearum* infection.

### 3.6. FgSna and lncRsn Negatively Regulate in F. graminearum

**Figure 6:**
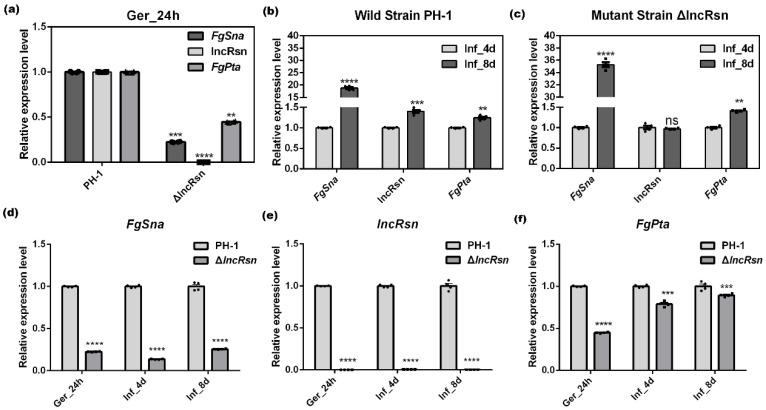
The expression pattern of FgPta, lncRsn, and FgSna during the nutrient mycelium period and the pathogenic period. (**a**) The expression level of lncRsn in the mutant; (**b**) The expression level of lncRsn and its neighboring genes in the wild-type PH-1 during the pathogenic period; (**c**) The expression level of lncRsn and its neighboring genes in the mutant strain ΔlncRsn during the pathogenic period; (**d**) The expression level of FgSna and its neighboring genes in the PH-1 and mutant strain ΔlncRsn during the nutrient mycelium period and the pathogenic period; (**e**) The expression level of lncRsn and its neighboring genes in the PH-1 and mutant strain ΔlncRsn during the nutrient mycelium period and the pathogenic period. (**f**) The expression level of FgPta and its neighboring genes in the PH-1 and mutant strain ΔlncRsn during the nutrient mycelium period and the pathogenic period, significant differences between the replicates are represented by **, ***, ****, no significant differences between the replicates are represented by ns.

lncRsn plays a regulatory role in modulating the transcription of its neighboring protein-coding genes, *FgSna* (upstream) and *FgPta* (downstream). Transcription of ncRNAs can have significant effects on flanking genes [51]. LncRNAs can regulate the transcription of neighboring genes, as demonstrated by the results (Figure 1c). lncRsn is located in the transcriptional termination regions of *FgSna* and *FgPta* and may be involved in their regulation by influencing their transcriptional termination. To further investigate this hypothesis, the gene expression levels of lncRsn, *FgSna*, and *FgPta* were examined during the nutrient mycelium period and the infection period (in wheat spikelets after 8 days of infection by different strains). The expression level of lncRsn in ΔlncRsn was close to zero compared with the wild-type strain (PH-1) during the nutrient mycelium period (Figure 6a), indicating that lncRsn had been successfully knocked out. Meanwhile, the expression levels of both *FgSna* and *FgPta* were decreased significantly, suggesting that lncRsn may positively regulate the expression of the upstream neighboring gene *FgSna* and the downstream neighboring gene *FgPta* during the nutrient mycelium and the infection period (Figure 6d–f). During the infection period, compared with the 4-day spikelet infected (Inf_4d) as the control, the results of the expression levels of each gene within the 8-day wheat spikelet infected from the wild-type strain showed that the expression of *FgSna* was up-regulated by 1778%, lncRsn was up-regulated by 40%, and *FgPta* was up-regulated by 24% (Figure 6b). Additionally, in the spikelet infected with the F. graminearum mutant strain on 8 days of infection, *FgSna* was up-regulated by 3426%, lncRsn by 40%, and *FgPta* by 24% (Figure 6c).

## 4. Discussion

Most lncRNAs are described in humans and other mammals [52]; in this study, we analyzed transcriptome data to screen for relevant lncRNAs during the infection period. To further investigate the effects of these lncRNAs on *F. graminearum* growth and pathogenicity, we constructed knockout vectors following the principle of homologous recombination and used PEG to mediate the transformation. The mutant strains obtained through PEG-mediated protoplast transformation were verified as in situ substitutions by RT-qPCR. In terms of nutrient mycelial growth, stress response, or cell wall integrity in *F. graminearum*, PH-1 strain, and ΔlncRsn are not significantly different. However, it led to significant differences in pathogenicity, conidial development, and sexual reproduction. Furthermore, in the yeast Saccharomyces cerevisiae, the deletion of an antisense lncRNA also affects sexual reproduction, similar to the results of the present study. This suggests that strains can still grow in the presence of deletions or mutations in antisense lncRNAs, which may represent a mechanism for biological evolutionary backup.

The rice blast fungus is unable to reproduce sexually under natural field conditions and instead relies on conidia for asexual production. In contrast, *F. graminearum* can reproduce through both asexual conidia and sexual ascospores, as these are crucial developmental stages in the *F. graminearum* life cycle and contribute to the pathogenesis of the disease. In the present study, the inoculation of wheat ears and sheaths, as well as young corn silks, with conidial suspensions of *F. graminearum* mutants revealed that a deletion mutant of lncRsn was significantly less pathogenic on wheat, although its ability to penetrate host tissues was not affected. SEM observations of the infection structures on wheat leaves indicated that the mutant formed a looser infestation structure compared with the wild-type strain, potentially explaining the reduction in pathogenicity. Additionally, the mycotoxin deoxynivalenol (DON), a major virulence factor of *F. graminearum*, plays an important role in the invasive growth of the fungal mycelium during wheat spike infection [53]. The absence of certain mutants interferes with the process of DON production and accumulation, further confirming the function of DON production in the virulence of pathogens. For instance, deletions of the genes *FgPEX1*, *FgPEX4*, and *FgCrz1A* lead to reduced DON production and virulence [54,55]. This suggests that lncRsn may indirectly affect DON biosynthesis and virulence by impacting the secondary metabolism of *F. graminearum*. Sexual reproduction in *F. graminearum* produces ascospores that are forcibly ejected into the air, playing an important function in surviving in adverse environments and the transmission of the disease [56,57,58]. The results of the current study reveal that the release of ascospores from lncRsn-deficient strains is significantly lower, indicating that lncRsn regulates the sexual reproduction stage of *F. graminearum*. The deletion of lncRsn severely affects the eruption of ascospores during sexual reproduction. Previous studies found that the buildup of turgor pressure within the extended asci is necessary for the forcible discharge of ascospores [59]. This turgor pressure is tightly related to the generation of ion fluxes, especially in K^+^, Na^+^, Cl^−^, and Ca^2+^ ion channels, as well as the accumulation of glycogen [57,58,60]. LncRsn appears to play a role in maintaining stress sensitiveness regulation in *F. graminearum*, as shown by the increased sensitivity of the mutant to various osmotic agents (0.5 M CaCl_2_, 0.2 M MgCl_2_, 1.2 M NaCl, and 1 M KCl) and cell wall integrity (CWI) assays. Three MAPK kinases (Mgv1, Gpmk1, and FgHog1. Mgv1) present in *F. graminearum* are responsible for maintaining cell wall integrity; *Gpmk1* is closely associated with sexual development and invasion processes, and *FgHog1* primarily mediates responses to osmotic stress and oxidative stress. Previous studies have indicated that these kinases may have overlapping functions in pathogenesis, sexual reproduction, and secondary metabolism [38]. 

LncRNAs typically exhibit shorter open reading frames, lower GC content, and fewer exons compared with protein-coding genes. Studies suggest that antisense lncRNAs may regulate the expression of neighboring genes in cis- or more distant genes in trans- through diverse mechanisms [52]. Protein-coding genes located within 10 kb upstream and downstream of lncRNAs are considered potential targets. Antisense lncRNAs are shown to control gene regulation at pre-transcriptional, transcriptional, and post-transcriptional levels via DNA–RNA, RNA–RNA, or protein–RNA interactions [61]. This review is centered on functional studies of antisense lncRNA-mediated regulation of neighboring gene expression [62]. Two lncRNAs were identified to target genes in cis, and to regulate pathogenicity and growth in fungal plant pathogens.UvlncNAT-MFS participates in the regulation of U.virens growth, conidiation, and various stress responses by forming RNA duplexes with UvMFS [63] *GzmetE-AS*, which is transcribed from the opposite strand of *GzmetE*, is involved in the asexual and sexual reproduction of *F. graminearum* by regulating the expression of *GzmetE* [16]. The antisense lncRNA *RNA5P* significantly influences the regulation of the *TRI5*, *TRI6*, and *TRI10* genes, which are important for virulence production in *F. graminearum* [17]. In this study, the antisense lncRNA lncRsn was located on the complementary strand of *FgSna*, transcribed in the opposite direction. *FgSna* expression was lower during pathogenicity and sexual reproduction compared with nutrient growth, contrasting the higher lncRsn levels during these periods. Deletion mutants showed significant differences from wild-type only during pathogenicity and reproduction, not nutrient growth, aligning with predictions. Quantitative PCR revealed that deleting lncRsn doubled *FgSna* expression in the late pathogenicity stage compared with early, suggesting lncRsn suppresses the neighboring *FgSna*. However, the downstream *FgPta* was unaffected, indicating that lncRsn transcription and processing do not impact *FgPta*. In subsequent backfill experiments, we simultaneously tested wild-type, mutant, and backfill strains for nutrient mycelial growth, germinal sheath, and cornhusk pathogenicity and found that the predicted results for the backfill strains were consistent with our expectations (Figures S3–S5). The results were similar among different deletion mutants, which was in line with our expectations. (Figure S6). These results suggest that lncRsn may reduce *F. graminearum* virulence, delay conidiation, disrupt ascospore eruption, and impair DON toxin synthesis by repressing *FgSna* and provide new insights into antisense lncRNA regulation of target genes.

## Data Availability

The data presented in this study are available on request from the corresponding author.

## Supplementary Materials

The following supporting information can be downloaded at: https://www.mdpi.com/article/10.3390/jof10100692/s1. Table S1: Primers used in this study; Table S2: Expression of each transcript at each period in *F. graminearum*; Table S3: Prediction of lncRsn coding potential with the help of CPC, CNCI and PLEK; Figure S1: the expression level of MAT genes in the PH-1 and ΔlncRsn during the sexual reproduction period; Figure S2: the expression level of TRI gene clusters in the PH-1 and ΔlncRsn after 7 days of TBI culture; Figure S3: Colony morphology of different strains on different plates; Figure S4: Comparison of pathogenicity of different strains on coleoptile; Figure S5: Comparison of pathogenicity of different strains on corn whiskers; Figure S6: Similar results were obtained in different deletion mutants.
