# Supplementary figures and images for: An Antisense Long Non-Coding RNA, LncRsn, Is Involved in Sexual Reproduction and Full Virulence in Fusarium graminearum
Source: J Fungi (Basel). 2024 Oct 3;10(10):692. doi: 10.3390/jof10100692 (PMC11508260; doi:10.3390/jof10100692)

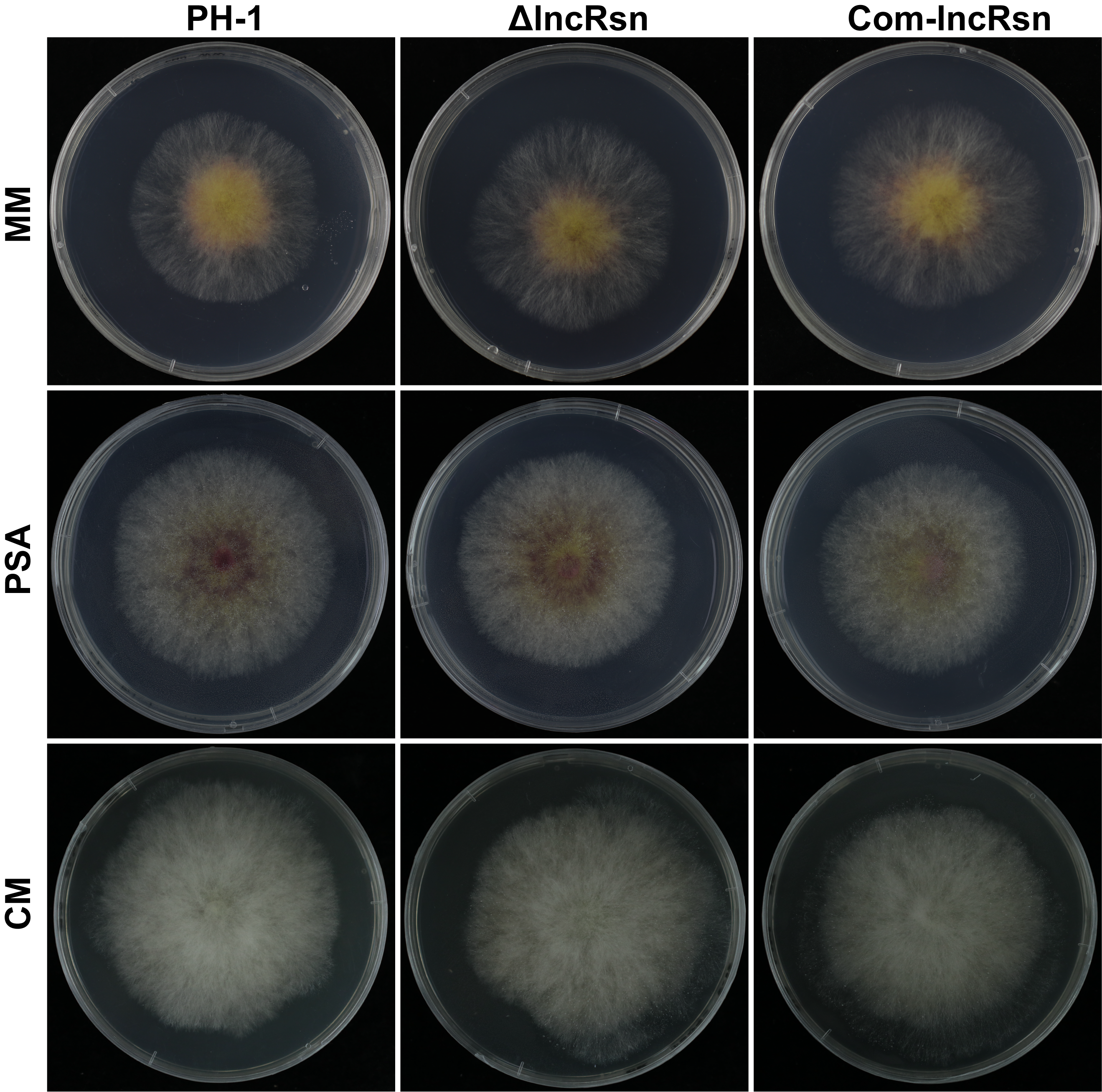

Supplement: Supplementary file 1 [file jof-10-00692-s001.zip › Figure S3.tif]

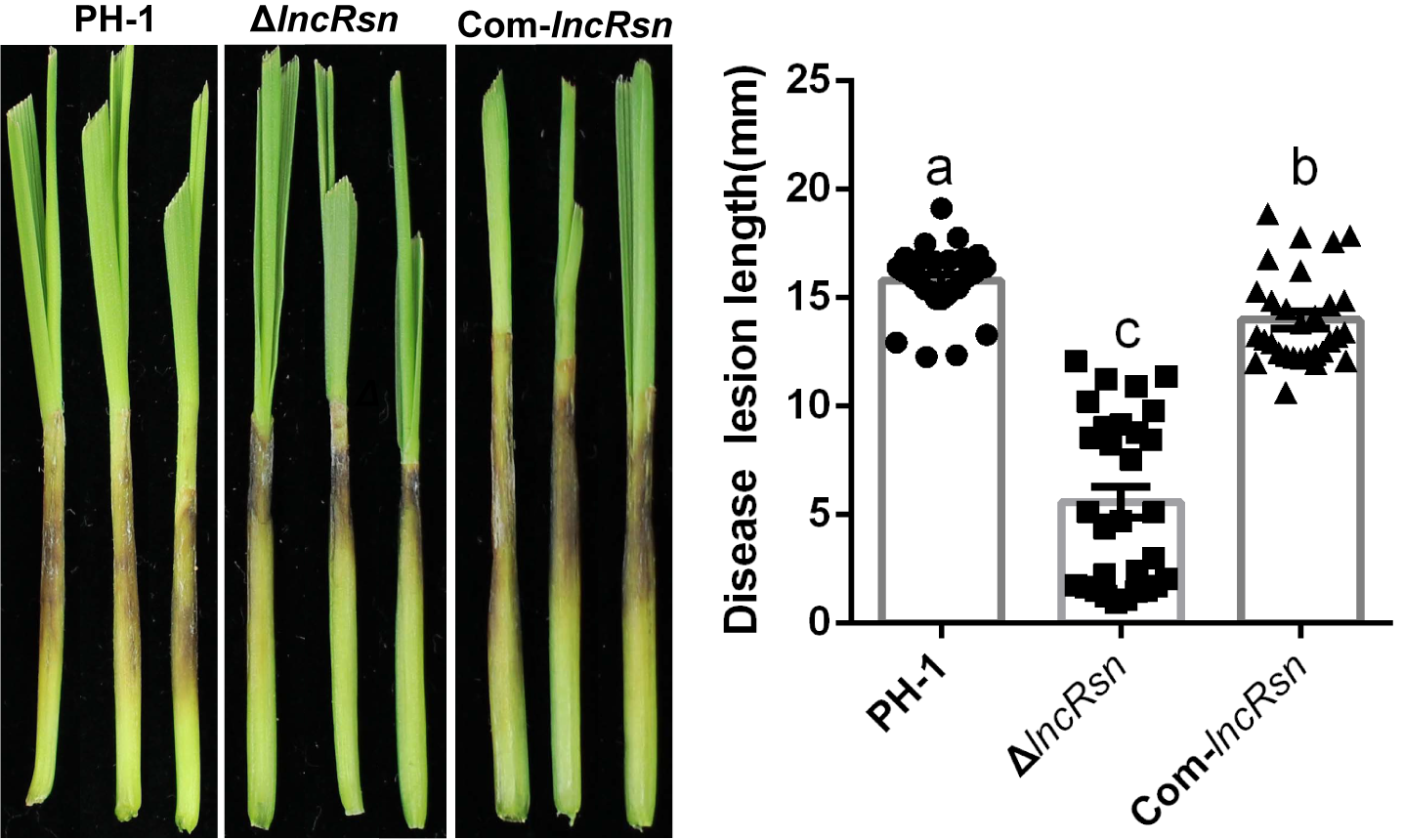

Supplement: Supplementary file 1 [file jof-10-00692-s001.zip › Figure S4.tif]

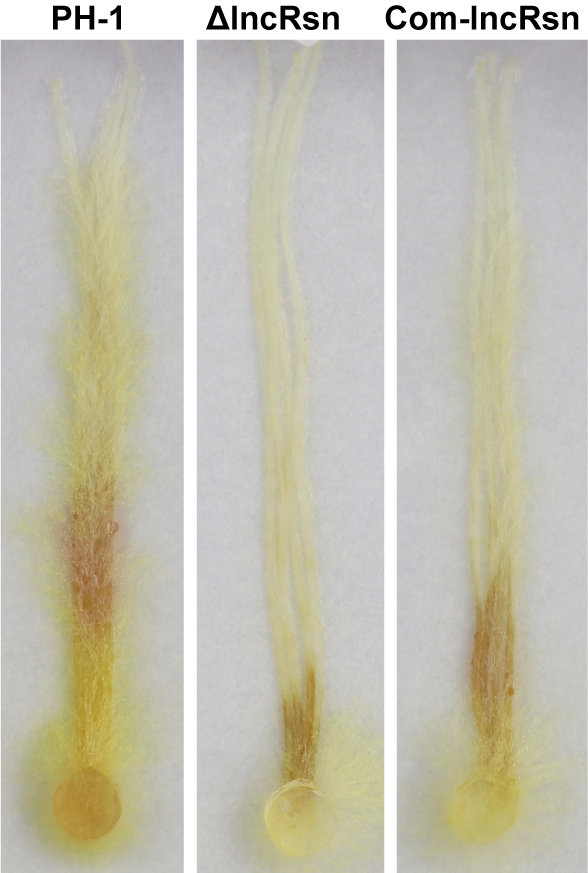

Supplement: Supplementary file 1 [file jof-10-00692-s001.zip › Figure S5.tif]
